# Preschoolers’ home music environment relates to their home literacy environment and parental self-efficacy

**DOI:** 10.1371/journal.pone.0313218

**Published:** 2024-11-07

**Authors:** Talia Liu, Helen Gray-Bauer, Kelsey E. Davison, Jennifer Zuk

**Affiliations:** Department of Speech, Language, and Hearing Sciences, Boston University, Boston, MA, United States of America; Leiden University: Universiteit Leiden, NETHERLANDS, KINGDOM OF THE

## Abstract

Positive relationships between the home literacy environment and children’s language and literacy development are well-established. However, existing literature has overlooked the potential contributions of the home music environment. Initial evidence indicates positive relationships between the home music environment and children’s emerging language and literacy skills, yet it remains unclear whether and how children’s home music and literacy environments may be related. Furthermore, parents’ sense of self-efficacy is known to impact the home environment provided for their children. Despite being linked with the home literacy environment, parental self-efficacy has not been directly investigated in relation to the home music environment. In the present study, 124 caregivers of preschoolers completed a one-time online survey about their children’s home music environment, home literacy environment, and parental self-efficacy. Partial correlations and hierarchical regressions reveal that children’s amount of music exposure is associated with qualitative (not quantitative) aspects of the home literacy environment, specifically parents’ use of interactive techniques during shared reading. Moreover, parental self-efficacy is associated with children’s amount of exposure to music. Overall, these findings support the need to further examine how the home music environment may meaningfully contribute to an enriching learning environment, especially to support language and literacy development.

## Introduction

Children’s home learning environments have been repeatedly shown to be predictive of children’s cognitive development and long-term academic outcomes [1–3]. Parental involvement in both general, day-to-day activities and domain-specific processes, such as engagement in literacy and/or numeracy-related concepts, are central to children’s home learning environments [4–7]. Within these home activities, parent engagement with young children is often informal, focusing on enjoyment and sheer exposure [8]. Parent-child engagement in the home can also be considered formal when parents intentionally target skill development in their activities [8]. Overall, parental involvement in both formal and informal engagement in domain-specific activities is known to contribute to the prediction of children’s cognitive development and long-term academic outcomes [5, 7, 8]. This home engagement is especially important for children at the preschool age, a pivotal time during which families support their children’s support their children’s readiness for formal schooling [9, 10]. Therefore, well-established literature suggests that fostering a high quality home learning environment lays a foundation for preschool-aged children’s long-term academic achievement.

The home *literacy* environment is one specific domain of the home learning environment that is especially important in fostering not only children’s cognitive development more broadly, but in facilitating early language and literacy development [7, 11–13]. In particular, parent engagement in shared reading activities with their children has been shown to positively relate to children’s emergent language and literacy outcomes [7, 14, 15]. Indeed, it has long been recognized that the home literacy environment, which encompasses both formal and informal aspects, provides enriched language exposure in early childhood [16, 17]. Despite the large body of evidence emphasizing the importance of shared reading, there is a need to further recognize that parent engagement in shared reading is only one aspect of children’s overall home learning environments that works in conjunction with other structural factors (e.g., stable aspects of the family background including socioeconomic status and parents’ educational beliefs) and other domain-specific parent-child activities. While research on children’s home learning environments, especially the home literacy environment, is well-established [1, 2, 7, 12, 18], one understudied domain in this context is the home *music* environment, which pertains to informal music interactions or engagement (e.g., singing and music-making) and musical exposure [19]. Although music is highly prevalent within children’s home environments and has been linked with language skills, the role of children’s home music environments in conjunction with other prominent aspects of children’s home learning environments, such as the home literacy environment, remains surprisingly underspecified. To address these missing links in existing literature, the present study seeks to address how the home music and literacy environments may be associated. Building an understanding of whether and how these domains may be associated serves as an important first step to conceptualizing the home music environment within the context of the broader home learning environment.

### Importance of the home literacy environment in supporting child development

Decades of research has established significant contributions of the home literacy environment to facilitating children’s language and emergent literacy development [14, 15]. Importantly, parent-child interactions during shared reading are known to involve more complex and rich language content than typically occurs in day-to-day conversations [16, 17]. Specifically, studies have shown higher adult word counts, more diverse vocabulary use, more conversational turns, and increased lexical complexity in parents’ spoken language during shared reading contexts than outside of shared reading contexts [16, 17, 20, 21]. Because shared book reading offers opportunities for rich language exposure, shared book reading interactions are implicated in supporting children’s language and literacy developmental trajectory [7, 11, 12, 14, 18].

The importance of parent-child shared reading, a central aspect of the home literacy environment, has been demonstrated in terms of both quantity and quality. Specifically, quantity of the home literacy environment is often characterized by the amount of time spent engaging in shared reading (i.e., duration) or the number of shared reading episodes (i.e., frequency) [22]. *Qualitative* aspects of the home literacy environment, on the other hand, capture *how* a parent approaches shared reading experiences with their child. This often encapsulates both informal and formal activities via parents’ use of interactive reading techniques, with formal strategies including print referencing, when a parent directs a child toward written language using strategies like pointing to print or asking questions about specific letters [23]. Another prominent aspect of shared reading strategies includes dialogic reading, which involves asking questions about the story or asking the child to fill in the blank, effective strategies that facilitate children’s active engagement in language expression during shared reading [24]. Both quantitative and qualitative aspects (including the use of print referencing and dialogic reading) of parent-child shared book reading have been shown to support children’s long-term language and literacy outcomes [7, 15, 17, 22, 25, 26].

Although the predictive value of the home literacy environment has been established, this factor by no means is solely indicative of children’s long-term outcomes; meta-analyses have revealed that book reading is estimated to predict only approximately 8% of variance in outcome measures involving language and emergent literacy gains, and reading achievement [14]. Certain structural, family-related factors have also been strongly linked with language and literacy development, particularly those pertaining to socioeconomic status [7, 27, 28]. Some evidence suggests that the home literacy environment may mediate the relationship between socioeconomic status and children’s literacy development [29, 30]. At the same time, even within low socioeconomic status groups, considerable variability in home literacy environments has been observed [11, 31]. Therefore, while collective evidence highlights the importance of the home literacy environment in facilitating children’s language trajectories, it is important to consider the role of other contributing factors.

### Parental self-efficacy is known to impact children’s home learning environments

While shared reading offers rich opportunities for fostering children’s language and emergent literacy skill development, it has been well-established that certain parental factors impact how often (quantity) and in what ways (quality) children receive these opportunities. In the context of children’s home environments, parental self-efficacy is one such factor associated with enriching learning practices and experiences parents provide for their children [32–35]. Parental self-efficacy refers to a parent’s perceived ability to be successful with parenting [36]. Regarding children’s home learning environments, parental self-efficacy has been shown to positively predict the *quantity* of children’s home learning experiences [35]. Specific to shared reading, parental self-efficacy is associated with not only shared reading quantity, but importantly also shared reading *quality*, specifically parents’ use of print referencing during a shared reading interaction with their child [22]. Complementary findings focused on parents with and without a history of language-based learning difficulties indicate that parents who engage more in shared reading and use interactive reading techniques more frequently with their children tend to have a greater sense of parental self-efficacy [37]. Importantly, parental self-efficacy has been linked with children’s school readiness and later academic performance (for review, see Albanese et al., 2019) [38]. Together, these relationships highlight the potential importance of parental self-efficacy in shaping children’s early home learning experiences.

### A need to better specify the role of music in children’s home learning environments

Music is a common source of input in young children’s home environments [19, 39, 40]. While the field has largely focused on characterizing children’s language exposure in everyday interactions, parents’ language use in these interactions is rich with “protomusical” elements involving melodic/pitch contours and rhythmic regularity (i.e., prosodic characteristics of child-directed speech) [41, 42]. Beyond the inherent musical elements of child-directed speech, parents also sing to and with their children [39, 40, 43]. Together, the ubiquity of home musical experiences and the linguistic content inherently embedded within parent singing makes informal musical experiences especially intriguing to better understand in the context of children’s learning environments. Thus, it is important to acknowledge the home music environment as a potential factor that may contribute to shaping the overall home environment and children’s subsequent developmental outcomes.

Both children’s music perception skills (e.g., pitch/rhythm discrimination) and formal experiences with music training have been positively linked with language and emergent literacy skills in early childhood [44–46]. Positive links between children’s musical skills/experience and several aspects of their oral language skills have been established (e.g., for recent reviews see Boorom et al., 2022 and Nayak et al., 2022) [47, 48], in domains including grammar and phonological awareness, a key predictor of children’s long-term literacy outcomes [49]. Meta-analyses of intervention studies revealed that preschool and school-aged children who received formal musical training (i.e., intervention groups) showed small but significant gains in phonological awareness skills [50] compared to both active and passive control groups. In a longitudinal investigation of 6–8 year old children, performance on a musical sequencing task was related to reading speed and accuracy [51] and predicted subsequent literacy outcomes [52]. In line with these findings, other studies have linked rhythm perception skills with early literacy skills including not only phonological awareness but also letter-sound knowledge [53] and print sensitivity [54]. Relatedly, intervention studies focused on struggling readers, specifically children with developmental dyslexia, suggest positive gains in reading abilities following participation in rhythm-based interventions [55, 56].

While relationships between language and formal music experience/abilities are widely studied and well-established, the role of *informal*, early childhood home music experiences in relation to language and literacy is less clear. Children’s informal home music environment has been characterized by music experiences, including exposure to musical sounds in recordings (e.g., toys, CDs, TV) or in live contexts, often through parent-child singing interactions [39]. Recognizing the inherent linguistic input embedded in parent singing, further investigation of this aspect of the home music environment is especially warranted in conjunction with other aspects of the home environment implicated in supporting children’s language development. Child-directed singing involves slower rates or tempos, exaggerated rhythms, and increased pitch variation compared to speech, which together help capture and maintain infants’ and children’s attention [57–59]. Together, these distinct characteristics of child-directed singing may make singing a vehicle to support language development [60]. Indeed, studies have shown that from as early as infancy, the overall home music environment and music engagement significantly relates to infants’ gestural communication and receptive vocabulary knowledge [61, 62]. At the preschool age, children’s home music environment has been positively associated with emerging language abilities, specifically phonological awareness and grammatical skills [63].

While the importance of the home language and literacy environment is well-established, scarcely any evidence has investigated the potential association between children’s home music and literacy environments. In one study examining a wide variety of home learning activities, reading to and singing with children daily showed significant effects (large and medium effects, respectively) on children’s concurrent language skills, specifically vocabulary knowledge [64]. While this prior study addresses the relative contributions of multiple home activities (including shared reading and singing) to children’s emerging language skills, it remains unclear to what extent these domain-specific activities may be associated with one another. Significant positive associations between children’s home music environment and other non-musical home experiences in early childhood have been indicated [40], yet only two studies to our knowledge have specified children’s home literacy experiences in relation to home music. One cross-sectional study indicated robust relationships between children’s home music and literacy environments in infancy, yet did not find any significant effects among preschool-aged children [19]. Of relevance to the present work, the home literacy environment was solely measured by an overall composite score summarizing both qualitative and quantitative aspects. The only other study to examine these relationships detected modest associations between quantitative aspects of toddler and preschoolers’ home music and literacy environments [65]. Therefore, it is conceivable that children’s home music and literacy environments may *jointly* underlie the rich language interaction known to contribute to facilitating children’s language development; however, more research is needed that directly evaluates children’s home music environments in relation to home literacy environments.

Moreover, parental self-efficacy is linked with the home literacy environment and the broader home learning environment, yet its relationship to the home music environment is less clear. Initial survey findings did not indicate relationships between children’s home music environment and parental self-efficacy [40, 66]; however, self-efficacy was only measured by a single question probing parents’ perceptions of how they fulfill the ‘global’ parent role (e.g., “not very good at being a parent”, “a very good parent”). This limited context in which self-efficacy has been examined to date highlights the need for more detailed characterization of self-efficacy (e.g., perceptions regarding their fulfillment of specific teaching- and play-related parenting tasks) [67] in relation to the home music environment. Overall, further investigation of the home music environment in relation to the home literacy environment as well as parental self-efficacy is essential to build our collective understanding of the potential role of music-related experiences in fostering enriching home learning environments for young children.

### Current study

To build a better understanding of potential associations between children’s home literacy environment, home music environment, and parental self-efficacy, the present study conducted a one-time online study with parents of preschool-aged children. The present study sought to determine whether parents who provide rich home literacy environments also provide rich home music environments for their children and further specify whether and how parental self-efficacy may be associated with each of these crucial components of children’s home environments. In line with initial evidence suggesting an association between the frequency of shared reading and music activities [65], we hypothesized that children’s home music environment will be positively associated with their home literacy environment. To our knowledge, this study is the first to differentiate and examine both quantitative and qualitative aspects of the home literacy environment in relation to the home music environment. In characterizing these differential aspects of the home literacy environment, this study enables investigation of which of these aspects are most closely associated with the home music environment. Moreover, based on existing research suggesting that parental self-efficacy is associated with children’s home literacy environments [22, 35, 37], we hypothesized that parental self-efficacy will also be associated with children’s home music environment (i.e., parents with a greater sense of self-efficacy will provide richer home music environments). By examining the home music environment in conjunction with both the home literacy environment and parental self-efficacy, the present study seeks to further our understanding of the role of the home music environment and factors that may contribute to parental involvement in providing their children with enriching home environments.

## Materials and methods

### Participants

Participants were 124 English-speaking parents of preschoolers aged 36–60 months old. The survey respondents were primarily mothers (83.1%, *n* = 103) with a mean age of 32.6 years old (*SD* = 5.93, *n* = 117). Parents were eligible to complete the survey if they were English-speaking, residing in the United States, over the age of 18, and had a child aged 36–60 months at the time of study participation. Families who completed the online study were based in 29 states representing all five geographic regions of the United States and the District of Columbia. See Table 1 for full respondent demographics. Participants were recruited through social media (e.g., Facebook) and word of mouth. Given broader goals of the research study, recruitment efforts targeted social media groups dedicated to advocacy and support for individuals and families with history of reading difficulties [37].

**Table 1:** Parent demographic information (*n* = 124).

	*n*	(%)
Race/Ethnicity		
Asian	3	(2.4)
Black or African American	6	(4.8)
Hispanic or Latinx	6	(4.8)
Multiracial	4	(3.2)
Native American or Alaska Native	2	(1.6)
Native Hawaiian or Pacific Islander	1	(0.8)
White	102	(82.3)
Household Income		
Less than $24,999	6	(4.8)
$25,000 - $49,999	22	(17.7)
$50,000 - $74,999	13	(10.5)
$75,000 - $99,999	28	(22.6)
$100,000 - $124,999	13	(10.5)
$125,000 - $149,999	10	(8.1)
$150,000 - $199,999	10	(8.1)
$200,000 - $249,999	4	(3.2)
$250,000 or more	11	(8.9)
Prefer not to respond	7	(5.6)
Highest Education Level		
8th grade or less	1	(0.8)
Some high school	10	(8.1)
High school diploma or equivalency (GED)	18	(14.5)
Apprenticeship	3	(2.4)
Some college	15	(12.1)
Two-year college degree or Associate’s degree (junior college or vocational school)	10	(8.1)
Four-year college degree or Bachelor’s degree	19	(15.3)
Some school beyond college	2	(1.6)
Graduate or professional degree	46	(37.1)

### Procedure

Data were collected from May to July 2021 via an online survey using REDCap, an online secure data server [68]. Survey items were designed to fall within or below a 4th to 5th grade Lexile level (determined by MetaMetrics [69]) in order to promote accessibility to parents. The survey was developed based on principles of universal design and was designed to be concise in accordance with recommendations from Goegan et al. [70]. The study received ethical approval from the Boston University Institutional Review Board. Participants completed an online consent form prior to starting the survey. The survey completion rate (ratio of users who finished the survey/users who completed the consent form) was 83%. Only survey responses with complete responses to all questions of interest pertaining to the home music and literacy environments were included in the present sample. No partial responses were acquired.

### Measures

#### Demographics

Demographic information captured by the survey included parents’ household annual income, highest level of education completed, race and ethnicity, and gender. Household annual income was rated on a nine-point scale from “Less than $24,999” to “$250,000 or more”, while highest level of education completed was rated on a nine-point scale from “8th grade or less” to “Graduate or professional degree”. A composite socioeconomic status score was calculated by averaging parents’ highest education level and household income.

#### Home music environment

The home music environment was characterized by two main factors: i) children’s overall amount of exposure to music or musical sounds on a typical day and ii) how often parents sing to their children per day. These two custom questions (Tables 2 and 3) were rated on ordinal scales. The amount of music exposure was rated on a 7-point scale from “Less than 5 minutes” to “Over 3 hours”, and parent singing was rated on a 7-point scale from “I do not typically sing with my child” to “Over 2 hours”.

**Table 2:** Survey distribution of the amount children are exposed to music.

	On a typical day, how often is your child exposed to music / musical sounds (e.g., recordings, radio, YouTube, music on television, live music-making, toys that make music)?	
	*n*	(%)
Less than 5 minutes	0	(0.0)
5–15 minutes	10	(8.1)
15–30 minutes (up to ½ hour)	48	(38.7)
31–60 minutes (up to 1 hour)	35	(28.2)
61–120 minutes (up to 2 hours)	16	(12.9)
121–180 minutes (up to 3 hours)	8	(6.5)
Over 3 hours	7	(5.6)

**Table 3:** Survey distribution of how often parents sing to their children.

	On a typical day, how often do you currently sing to your child?	
	*n*	(%)
I do not typically sing with my child	22	(17.7)
1–15 minutes	54	(43.5)
15–30 minutes (½ hour)	35	(28.2)
31–45 minutes	9	(7.3)
46–60 minutes (1 hour)	2	(1.6)
61–120 minutes (2 hours)	2	(1.6)
Over 2 hours	0	(0.0)

#### Home literacy environment

To characterize the home literacy environment, parents responded to questions addressing both the quantity and quality of shared reading. Shared reading quantity was captured by two separable variables: i) the amount of shared reading time per week and ii) access to children’s books (including printed books, e-books, and audiobooks). Shared reading quality was measured by how often parents use common interactive reading techniques when reading to their children. More detailed descriptions of these shared reading variables are as follows:

For the amount of shared reading time, parents responded to four questions about how many days per week they read bedtime stories to their children, how many times per week they read to their children, the duration of typical reading sessions, and how much time per week they spend reading with their child. These questions were adapted from Puglisi et al. [71] and rated on 5-point ordinal scales. Responses from these questions were averaged to create a composite score for the overall amount of shared reading time per week.

For access to children’s books, parents responded to three questions about how many children’s books they have at home (not including e-books), how many e-books their child has been exposed to, and how much time their child spends listening to audiobooks per week. These questions were rated on 5-point Likert scales and averaged to create a composite score for children’s access to children’s books.

Parents reported how frequently they used the following interactive reading techniques: asking questions about the story, pointing out letters and words, practicing the names or sounds of letters, having the child fill in words at the end of a phrase, doing an activity based on the story, and practicing rhyming (adapted from Radville et al.) [72]. Each interactive reading technique was rated on a 5-point Likert scale ranging from “never” to “about every day”. Survey items were ranked on an ordinal scale. In addition to assessing individual reading techniques, we computed a composite score for the overall use of interactive reading techniques by averaging responses across all techniques.

#### Parental self-efficacy

Parents’ perceived self-efficacy was measured by adapting a subset of statements from the “Teaching” domain of the Self-Efficacy for Parenting Tasks Index–Toddler Scale (SEPTI-TS) [67]. Statements were modified in order to meet a 4th-5th grade Lexile level and to achieve consistency with respect to the scale’s directionality across all statements. Parents responded to a total of six statements (see Supporting Information) by rating their agreement to each statement on a six-point Likert scale from “Strongly Disagree” to “Strongly Agree”. The sum of these ratings (possible range: 6–36) resulted in a total score for parental self-efficacy, where a higher score indicated stronger self-efficacy.

### Analyses

To address the first aim of the study of whether parents who provide their children rich home literacy environments also provide rich home music environments, we conducted Spearman’s rho rank-order partial correlations between the home music environment and home literacy environment variables. To address the second research aim of examining relationships between the home environment and parental self-efficacy, we conducted partial Spearman’s correlations between the home environment variables (both music and literacy) and parental self-efficacy. Due to known associations between family socioeconomic status and children’s home environments, including the home literacy environment [13, 73], composite socioeconomic status scores were incorporated as a covariate in all partial correlations employed. Thereafter, False Discovery Rate (FDR) correction was used to control for multiple comparisons [74]. FDR correction is a favorable approach to correction for multiple comparisons as it controls for the expected proportion of false positives amongst rejected hypotheses, thereby providing a less stringent approach compared to more conservative options, such as Bonferroni correction, which can be susceptible to false negatives (i.e., Type II errors) [63]. Here, we focus our interpretation on significant effects (i.e., *p* < .05) after FDR correction.

In a final step, hierarchical linear regressions were conducted in order to specify the relative associations through simultaneous consideration of how key variables are associated with the home music environment. Regression models were built with key variables of interest–socioeconomic status, aspects of the home literacy environment, and parental self-efficacy– as predictors, and the home music environment as the outcome variable of interest. Variables were entered into the hierarchical linear regressions in the following order: socioeconomic status (composite of parent education and income), parental self-efficacy, access to children’s books, shared reading time, and interactive reading techniques. Socioeconomic status was entered first as a macro-level environmental factor that broadly influences families’ resources, opportunities, and ability to engage in home activities [75, 76]. Parental self-efficacy was added second because it is a factor known to influence the home learning environment parents provide [32]. Next, access to children’s books was added to the model, as it is a prerequisite for engaging in shared reading. Thereafter, the amount of time spent engaging in shared reading was added to the model, as the frequency and duration of shared reading provide the necessary platform for engaging in high quality interactions. Lastly, the use of interactive reading techniques, which represent the richness of shared reading interactions, was added to the final model. Therefore, the final regression model revealed how the home music environment is associated with aspects of the home literacy environment and parental self-efficacy, while concurrently accounting for all variables of interest, including socioeconomic status.

## Results

### Overall characterizations of the home music environment

All parents reported that their child is exposed to music or musical sounds for at least 5 minutes within a typical day, with a median endorsement indicating up to 31–60 minutes of music exposure per day (Table 2). Eighty-two percent of parents endorsed singing to their children for at least 5 minutes on a typical day, with a median response indicating 1–15 minutes per day spent singing to their children (Table 3).

### Overall characterizations of the home literacy environment

**Fig 1:**
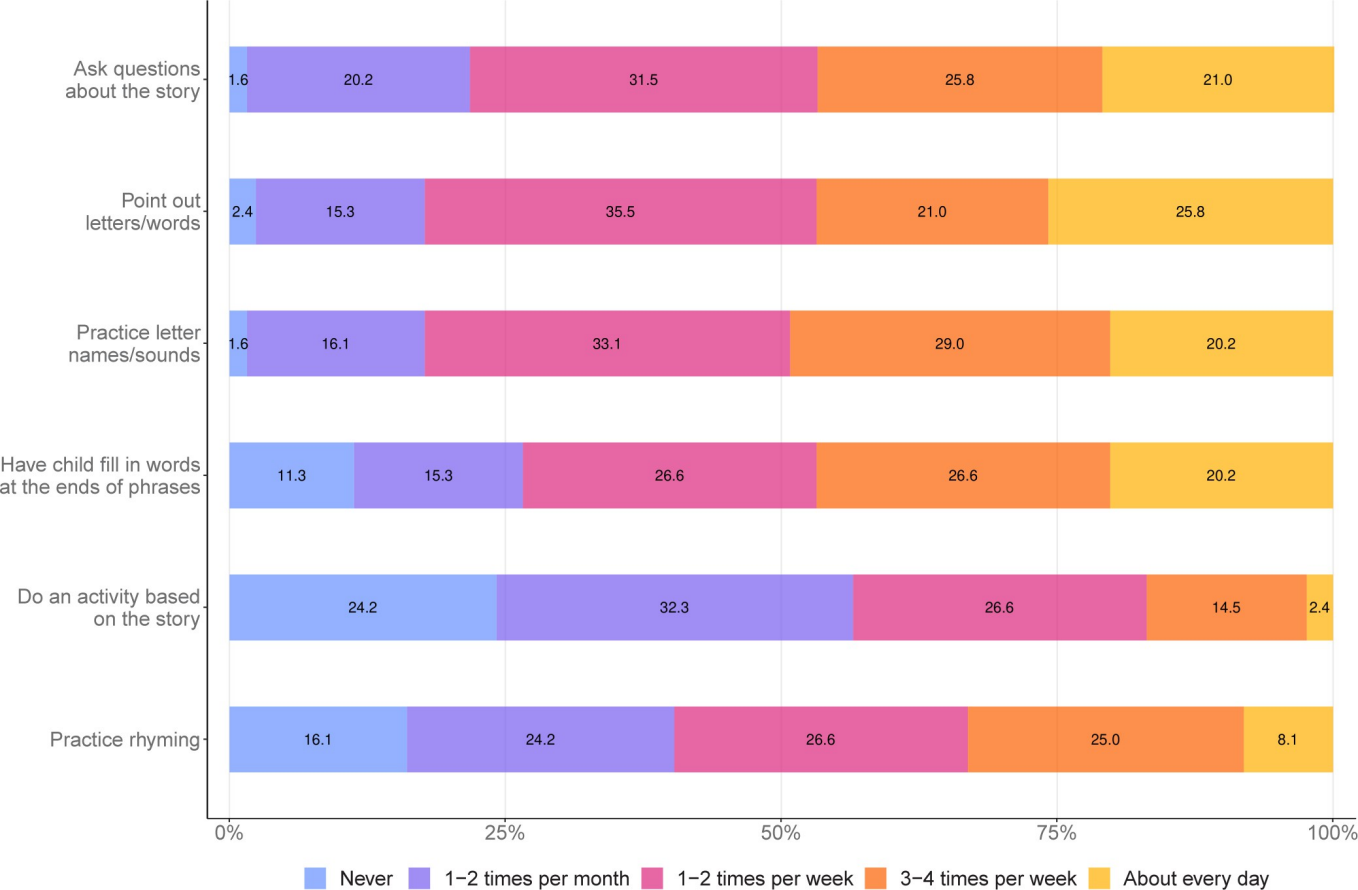
Distribution of responses to how often parents use interactive shared reading techniques.

An overview of the distribution of responses to individual survey questions addressing quantitative aspects of the home literacy environment can be found in Table 4. For a visual summary of parents’ use of interactive reading techniques within the present sample, see Fig 1. Response medians and modes for the three composite home literacy environment variables can be found in S1 Table.

**Table 4:** Distribution of responses to individual survey questions regarding quantitative aspects of the home literacy environment.

	*n*	(%)
**In a typical week, how often do you read bedtime stories to your child?**		
I do not typically read bedtime stories with my child	5	(4)
1–2 days	37	(29.8)
3–4 days	24	(19.4)
5–6 days	17	(13.7)
7 days	41	(33.1)
**Other than bedtime, how many times each week do you read to your child?**		
Fewer than 2 times	19	(15.3)
2–4 times	60	(48.4)
5–7 times	28	(22.6)
8–10 times	8	(6.5)
More than 10 times	9	(7.3)
**On average, how long are your reading sessions with your child?**		
10 minutes or less	24	(19.4)
About 11–20 minutes	64	(51.6)
About 21–30 minutes	25	(20.2)
About 31–40 minutes	9	(7.3)
Longer than 40 minutes	2	(1.6)
**On average, how much time do you spend reading with your child each week?**		
I do not typically read with my child	1	(0.8)
Less than 2 hours	52	(41.9)
2–6 hours	51	(41.1)
6–12 hours	18	(14.5)
More than 12 hours	2	(1.6)
**About how many children’s books do you have at home (not including e-books)?**		
Fewer than 10	3	(2.4)
10–25	19	(15.3)
26–50	47	(37.9)
101–200	27	(21.8)
201–400	19	(15.3)
More than 400	9	(7.3)
**About how many children’s e-books has your child been exposed to?**		
Fewer than 10	52	(41.9)
10–25	33	(26.6)
26–50	19	(15.3)
101–200	11	(8.9)
201–400	7	(5.6)
More than 400	2	(1.6)
**On average, how much time does your child spend listening to audiobooks each week?**		
0 hours	53	(42.7)
Less than 1/2 hour	19	(15.3)
1/2 hour-1 hour	29	(23.4)
1–2 hours	16	(12.9)
2–4 hours	2	(1.6)
More than 4 hours	5	(4)

### Children’s home music environment in relation to the home literacy environment

**Fig 2:**
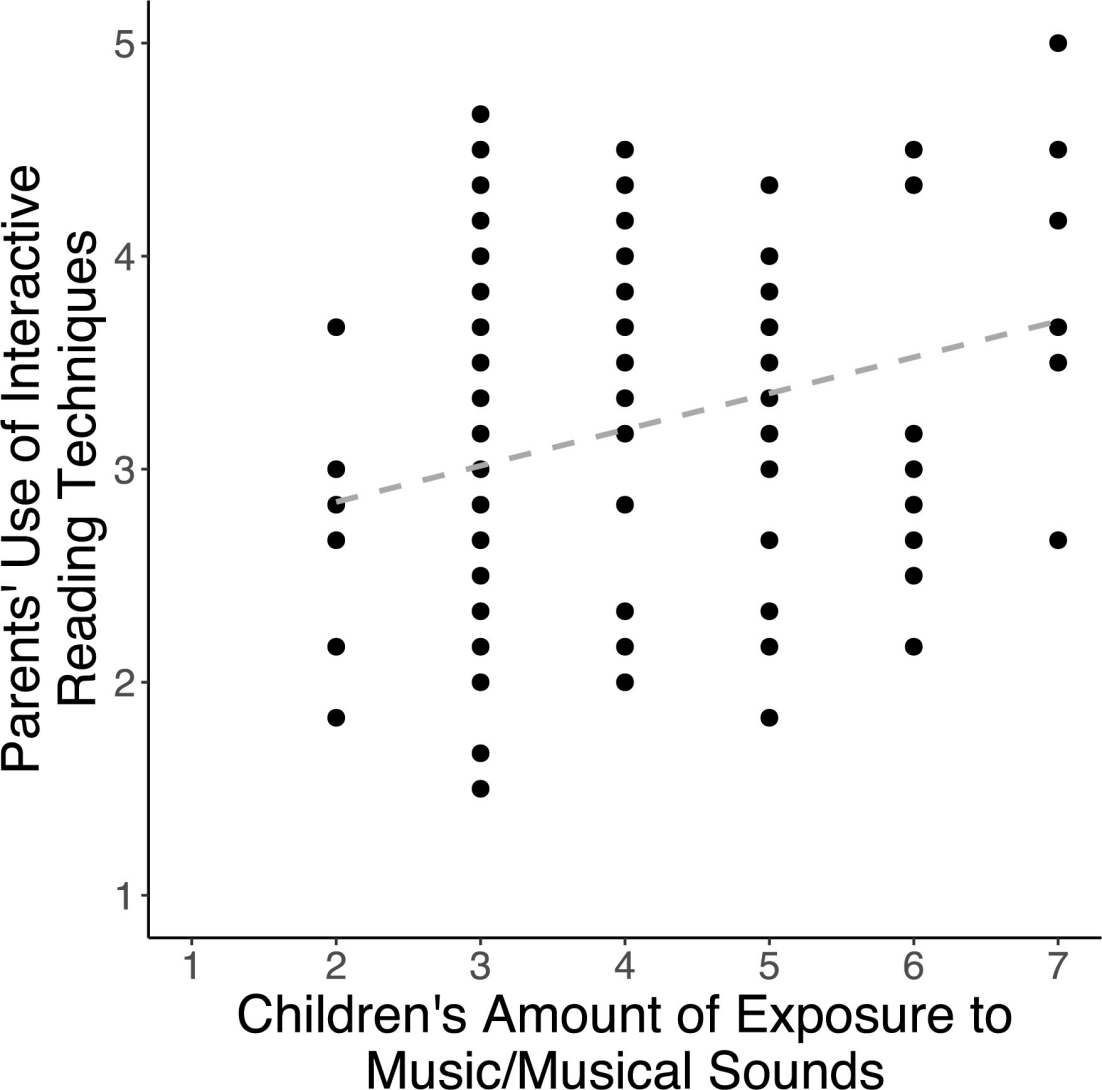
Children’s amount of music exposure is associated with parents’ use of interactive reading techniques. *Note*: The correlation plot depicts the raw relationship between variables and does not reflect the partial correlation controlling for socioeconomic status. A trend line is included to indicate the direction of the correlation.

Partial Spearman correlations, controlling for socioeconomic status, revealed positive associations between children’s amount of music exposure and the frequency of interactive reading techniques parents use during shared book reading [*r*_s_(124) = 0.247, *p* = .011] (Fig 2, Table 5). To further investigate the role of interactive reading techniques in relation to the home music environment, post hoc partial correlations were conducted between individual techniques and the home music environment variables. Of the individual interactive reading techniques, amount of music exposure was related to how often parents indicated that they ask questions about the story [*r*_s_(124) = 0.322, *p* < 0.001], point out letters and words [*r*_s_(124) = 0.264, *p* = .005], and practice rhyming during shared book reading experiences [*r*_s_(124) = 0.230, *p* = .015] (Table 6). The amount of exposure to music or musical sounds was not related to overall scores for the amount of shared reading time per week or access to children’s books.

**Table 5:** Correlation table of primary measures of interest.

	Home music environment variables	
Home literacy environment and parental self-efficacy variables	Exposure to music/musical sounds	Time spent singing to child
Shared reading time	0.089	0.049
Interactive reading techniques	0.247*	0.162
Access to children’s books	0.018	-0.037
Parental self-efficacy	0.273**	0.112

Note

*p < .05

**p < .01

***p < .001. *p*-values after FDR correction

**Table 6:** Correlations between home music environment variables and interactive reading techniques.

	Home music environment variables	
Interactive reading technique	Exposure to music/musical sounds	Time spent singing to child
Ask questions about the story	0.322***	0.028
Point out letters/words	0.264**	0.066
Practice letter names/sounds	0.131	0.091
Have the child fill in words at the end of a phrase	0.065	0.113
Do an activity based on the story	0.082	0.291**
Practice rhyming	0.230*	0.157

Note

*p < .05

**p < .01

***p < .001. *p*-values after FDR correction

The amount of time parents sing to their children did not demonstrate any significant associations with the overall indicators of the home literacy environment (shared reading time, amount of interactive reading techniques used, or access to children’s books). However, when examining relationships with specific interactive techniques, parents’ time spent singing to their children was positively associated with how often parents did an activity based on the story during shared reading interactions [*r*_s_(124) = 0.291, *p* = 0.002]. For a full table of bivariate Spearman’s rho rank-order correlations with all variables, see S2 Table. Notably, comparable associations between home literacy and home music variables were observed across both bivariate and partial correlations controlling for socioeconomic status.

### Parental self-efficacy in relation to children’s home music and literacy environments

**Fig 3:**
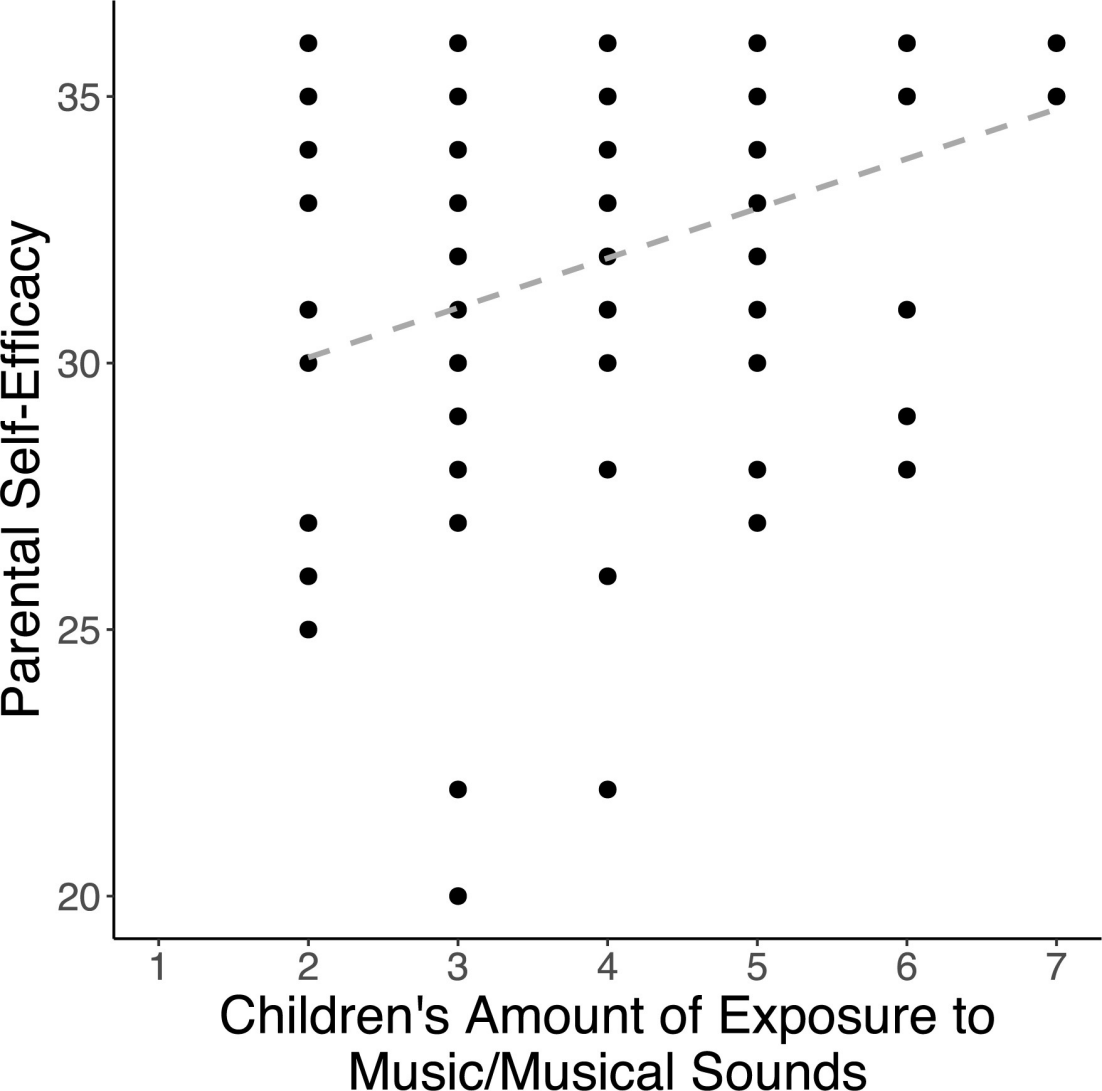
Children’s amount of music exposure is associated with parental self-efficacy. *Note*: The correlation plot depicts the raw relationship between variables and does not reflect the partial correlation controlling for socioeconomic status. A trend line is included to indicate the direction of the correlation.

Specific associations between parental self-efficacy and music and literacy-related aspects of the home environment were observed. In terms of children’s home music environment, a significant positive correlation between parental self-efficacy and children’s amount of exposure to music or musical sounds was observed [*r*_s_(124) = 0.273, *p* = .007] (Fig 3), but there was no association between parental self-efficacy and the amount of time parents spent singing to their children [*r*_s_(124) = 0.112, *p* = .297]. Regarding the home literacy environment, significant positive correlations were indicated between parental self-efficacy and the overall amount of shared reading time [*r*_s_(124) = 0.369, *p* < 0.001] as well as how often parents used interactive reading techniques [*r*_s_(124) = 0.268, *p* = .007]. Parental self-efficacy was not significantly related to access to children’s books [*r*_s_(124) = 0.123, *p* = .263].

### Concurrent investigation of the respective associations between the home literacy environment, parental self-efficacy, and home music environment

Given the significant correlations observed between the home literacy environment, parental self-efficacy, and the home music environment–specifically, children’s exposure to music–hierarchical linear regressions were employed to concurrently examine the respective associations between these variables of interest (for a full summary of the hierarchical linear regression model, see Table 7). In Model 1, with socioeconomic status as the sole predictor, socioeconomic status did not significantly account for variability in children’s music exposure (*R*^2^ = 0.026, *R*^*2*^_*adj*_ = .018, *p* = .071). When parental self-efficacy was added into the model (Model 2), parental self-efficacy was shown to significantly account for an additional 8.2% of the variability in children’s music exposure, *F*(2,121) = 7.35, *p* < .001 (*R*^2^ = 0.108, *R*^*2*^_*adj*_ = 0.094, *p =* .001). In the two subsequent models (Models 3 and 4), adding access to children’s books and shared reading time did not significantly account for additional variability in children’s music exposure (Model 3: *R*^2^ = 0.108, *R*^*2*^_*adj*_ = 0.086, *p* = .999; Model 4: *R*^2^ = 0.109, *R*^*2*^_*adj*_ = 0.079, *p* = .750). However, in the final model (Model 5), which included socioeconomic status, parental self-efficacy, and all three factors of the home literacy environment (including the addition of parents’ use of interactive reading techniques) as predictors, both parental self-efficacy and parents’ use of interactive techniques significantly contributed to variance in children’s music exposure, *F*(5,118) = 4.22, *p* = .001 (*R*^2^ = 0.152, *R*^*2*^_*adj*_ = 0.116, *p* = .001). In this final model, adding parents’ use of interactive techniques accounted for 4.2% of additional variability in children’s music exposure, resulting in a final model that explained 15.2% of variance in children’s music exposure. These findings were stable when music exposure and parent singing scores were averaged together to create a composite for the home music environment.

**Table 7:** Model summaries of hierarchical linear regressions predicting children’s music exposure.

	Model 1		Model 2		Model 3		Model 4		Model 5	
Predictors	β	*t*	β	*t*	β	*t*	β	*t*	β	*t*
Intercept	3.38	11.39***	0.28	0.28	0.28	0.27	0.29	0.29	-0.02	-0.02
Socioeconomic status (composite)	0.09	1.82	0.02	0.43	0.02	0.43	0.02	0.33	0.03	0.62
Parental self-efficacy			0.11	3.33**	0.11	3.30**	0.11	3.02**	0.09	2.63**
Access to children’s books					0.00	0.00	-0.02	-0.12	-0.08	-0.58
Shared reading time							0.06	0.32	-0.08	-0.40
Interactive reading techniques									0.39	2.43*
Model F-test	*F*(1,122) = 3.31		*F*(2,121) = 7.35***		*F*(3,120) = 4.86**		*F*(4,119) = 3.64**		*F*(5,118) = 4.22**	
*R*^*2*^ / *R*^*2*^ *adjusted*	0.026 / 0.018		0.108 / 0.094		0.108 / 0.086		0.109 / 0.079		0.152 / 0.116	
*R*^*2*^ Δ	0.026		0.082**		0.000		0.001		0.042*	

Note

*p < .05

**p < .01

***p < .001.

## Discussion

The present study provides novel insights into how children’s home music environment relates to their home literacy environment and how both of these environmental factors are associated with parental self-efficacy. This study builds on a large body of existing literature documenting the importance of an enriching home literacy environment in supporting children’s development [7, 11, 71] by acknowledging how children who are exposed to a more enriching home literacy environment tend to also be exposed to more music. Interestingly, the present findings reveal that children’s amount of exposure to music is specifically associated with qualitative over quantitative aspects of the home literacy environment, particularly parents’ use of interactive techniques during shared reading. These results suggest that beyond the sheer quantity of exposure, children exposed to more enriching musical experiences at home also tend to have parents who are engaging in more interactive reading techniques during shared reading. Moreover, parental self-efficacy is associated with both children’s amount of exposure to music as well as quantitative and qualitative aspects of shared reading. It is important to note that all findings are indicated even when controlling for socioeconomic factors. Taken together, the present study provides a promising foundation for future work to further investigate the utility of informal home musical experiences for promoting early childhood development.

The present findings extend limited existing literature by utilizing, for the first time, a more granular measure that differentiates quantitative and qualitative aspects of the home literacy environment in relation to home music environment. Whereas the previous findings either only looked at the quantity of the home literacy environment [65] or a composite home literacy environment score (as measured by the StimQ-Reading subscale) [19], the associations indicated in conjunction with results from the hierarchical regressions suggest that music exposure is linked with *qualitative* over quantitative aspects of the home literacy environment. This novel finding indicates that parents who use shared reading techniques more frequently, and therefore tend to provide their children with more enriching shared reading experiences, also tend to provide their children with more musical experiences. Importantly, concurrently accounting for quantitative aspects of the home literacy environment, parental self-efficacy, and socioeconomic status in the hierarchical regressions revealed significant effects of specifically parents’ use of interactive techniques in relation to musical exposure. When investigating associations between the home music environment and specific shared reading techniques (i.e., qualitative aspects), children’s amount of music exposure was positively associated with more frequent use of asking questions about the text, practicing letter/word sounds, and practicing rhyming. These interactive techniques are related to the facilitation of both written language awareness and phonological awareness, foundational domains of emergent literacy skills [23]. Importantly, the relationships presented here do not suggest that the quality of home activities matters more than the quantity, nor does it suggest any influence of one activity (i.e., music or literacy) on the other. Rather, the present study serves as a first step to suggest that the home music environment may be particularly associated with qualitative aspects of the home literacy environment.

As a common home activity that infuses linguistic content, parent singing is one particularly relevant component of children’s home music environments that has potential to impact children’s language development. In the present study, the amount parents sing to their children was specifically only associated with how often parents report engaging in an activity based on a story in shared reading contexts, a technique involving extratextual talk that may help facilitate listening and story comprehension [77, 78]. This association provides initial evidence that parents who sing more to their children may also provide enriching and engaging shared reading interactions. It is interesting to acknowledge such a specific association with parent singing indicated relative to the robust associations between the overall home music environment and numerous qualitative aspects of shared reading. The distinction in these findings with parent singing versus overall music exposure could be at least partly attributed to reported declines in parent singing from infancy to the toddler and preschool years [43, 79], whereas no decline in the amount of music exposure provided was observed [43]. Regardless of differences in parent singing between infancy and the preschool years, parent singing toward preschoolers tends to add more informational content (e.g., through the emphasis of key words) [80] and therefore may be an avenue through which parents can enrich their children’s home environments.

In addition to examining relationships between children’s home music and literacy environments, this study expands on recent literature characterizing links between parental self-efficacy and the quantity and quality of the home literacy environment [22, 37] by illuminating how these factors are also associated with the home music environment. Positive associations between parental self-efficacy and children’s exposure to music or musical sounds revealed by the present study suggest that parents who feel more confident in their ability to teach their children about the world and new concepts may also tend to expose their children to more music. Importantly, these associations were significant even when controlling for parental education and household income. Moreover, hierarchical linear regressions suggest significant effects of parental self-efficacy over and above socioeconomic status in the present sample. Considering the finding that increased parental engagement during shared reading (i.e., through the use of interactive techniques) was associated with more musical exposure, it is not surprising that parental self-efficacy was also associated with both of these factors. As parental self-efficacy can impact how much parents interact and engage with their child, it is important to consider self-efficacy and other relevant parental factors when examining children’s home environments.

### Limitations and future directions

The present survey study offers a first step toward understanding the role of the home music environment within the broader home learning environment by providing foundational evidence of significant associations between the home music environment, the home literacy environment, and parental self-efficacy. These findings are to be interpreted in light of several limitations, which present important opportunities for future research. While a strength of the present study is to examine the relevance of parental self-efficacy when accounting for key aspects of socioeconomic factors (family income and parent education), it is essential that future research examining children’s home environments does not overlook the potential contributions of other parental and family factors and experiences. Importantly, the cross-sectional survey design precludes any causal interpretations of the present work. Future research involving longitudinal and/or intervention study designs is essential in order to determine how children’s home music and literacy environments and parental self-efficacy interact with one another. While the hierarchical linear regressions serve as an initial step toward a more comprehensive understanding of how these variables are associated, future research that directly measures children’s language and emergent literacy skills is needed to further our understanding of how children’s home music environment may be related to their language and literacy skill development. Furthermore, more fine-grained and detailed characterization of the formality of home activities is needed to examine the relative importance of specific strategies. For example, effects of the home environment on children’s development may in part be due to more formal, teaching-oriented activities, such as practicing letter names and sounds [81]. Longitudinal study designs are especially needed in order to build our understanding of the predictive role of the home music environment in relation to children’s subsequent outcomes.

Future work is needed to address the likelihood that some home music and literacy activities may co-occur in children’s everyday environments [65]. The use of naturalistic measurements, such as home audio recordings [39] or home observations of shared reading interactions [25], would not only enable more robust characterization of the quantity and quality of parents’ engagement in home music and literacy-related activities but also uncover the extent to which music or singing is used *within* shared reading interactions in real-time (as is suggested by some existing literature [82]). This would help illuminate the utility of combining activities to facilitate skill development. Incorporating song-based picture books into shared reading activities provides increased opportunities for shared engagement and can foster bonding and joyful experiences for parents and their children [83, 84], as parent-child musical activities are familiar, predictable, and reinforcing to many children [85].

Finally, it is notable that controlling for socioeconomic status did not change the main effects observed in initial bivariate correlations, nor did socioeconomic status significantly contribute to the variance in children’s music exposure in the present sample. The current sample includes families with a range of socioeconomic backgrounds, representing all income levels. However, survey respondents predominantly identify as White and as holding graduate or professional degrees. Therefore, the present findings are not generalizable to all families in the United States and are to be interpreted with this in mind. Future work that examines the home music and literacy environments and includes families with diverse cultural, linguistic, and socioeconomic backgrounds is needed.

## Conclusions

Overall, the present study has established significant associations between specific aspects of preschool-aged children’s home music environment, home literacy environment, and parental self-efficacy. Specifically, children’s amount of music exposure was associated with both parents’ use of interactive techniques during shared reading and parental self-efficacy. The present study lays the groundwork for future work to further examine the mechanisms by which music exposure and/or engagement in musical activities in early childhood may shape children’s long-term language and literacy skill development. Therefore, the present findings support a growing body of evidence suggesting the potential utility of music and song in conjunction with or perhaps even during shared reading to support children’s learning and literacy skill development. Furthermore, building our understanding how parental self-efficacy relates to parents’ involvement in home music and literacy activities has potential to inform approaches to supporting parents in fostering their children’s development. Given the prevalence of music in children’s early lives, this work provides more evidence to support the need for future work to more directly address the under-looked home music environment as a meaningful and enriching part of children’s home environments.

## Supporting information

S1 Appendix Parental self-efficacy questions. (PDF)

S1 Table Survey medians and modes for the quantity and quality of the home literacy environment (possible range = 1–5). (PDF)

S2 Table Bivariate correlations between the home music environment, home literacy environment (including specific interactive techniques, parental self-efficacy, and composite socioeconomic score (SES). (PDF)
