# Supplementary material for: Preschoolers’ home music environment relates to their home literacy environment and parental self-efficacy
Source: PLoS One. 2024 Nov 7;19(11):e0313218. doi: 10.1371/journal.pone.0313218 (PMC11542833; doi:10.1371/journal.pone.0313218)
Supplement: S1 Appendix — (PDF) [file pone.0313218.s001.pdf]

S1 Appendix. Parental Self-Efficacy Questions.

All statements were rated on a 6-point Likert scale: “Strongly Disagree”, “Disagree”, “Somewhat Disagree”, “Somewhat Agree”, “Agree”, “Strongly Disagree”.

1. I believe my child learns a great deal from my efforts to teach them about life.
2. I am confident in my ability to help my child with learning new concepts and words.
3. I easily figure out the right level of instruction when I’m trying to explain something to my child.
4. I easily find opportunities to teach my child things about the world when we are together.
5. I feel confident helping my child learn more about their environment.
6. I am confident in my ability to read books with my child.
