# Supplementary material for: Preschoolers’ home music environment relates to their home literacy environment and parental self-efficacy
Source: PLoS One. 2024 Nov 7;19(11):e0313218. doi: 10.1371/journal.pone.0313218 (PMC11542833; doi:10.1371/journal.pone.0313218)
Supplement: S1 Table — (PDF) [file pone.0313218.s002.pdf]

S1 Table. Survey medians and modes for the quantity and quality of the home literacy environment (possible range = 1-5).

	Median	Mode
Amount of shared reading time per week	2.63	3.25
Use of interactive reading techniques	3.17	2.83
Access to children’s books	2.33	2.33
