# Supplementary material for: Preschoolers’ home music environment relates to their home literacy environment and parental self-efficacy
Source: PLoS One. 2024 Nov 7;19(11):e0313218. doi: 10.1371/journal.pone.0313218 (PMC11542833; doi:10.1371/journal.pone.0313218)
Supplement: S2 Table — (PDF) [file pone.0313218.s003.pdf]

S2 Table. Bivariate correlations between the home music environment, home literacy environment (including specific interactive techniques, parental self-efficacy, and composite socioeconomic score (SES).

	1.	2.	3.	4.	5.	6.	7.	8.	9.	10.	11.	12.
1. Exposure to music/musical sounds												
2. Time parent sings to child	0.219*											
3. Amount of shared reading time	0.140	-0.030										
4. Use of interactive techniques	0.256**	0.140	0.384***									
5. Access to written texts	0.015	-0.033	0.349***	0.321***								
6. Ask questions about the story	0.352***	-0.036	0.472***	0.644***	0.105							
7. Point out letters and words	0.288**	0.020	0.366***	0.783***	0.237*	0.552***						
8. Practice the names or sounds of letters	0.142	0.072	0.246*	0.733***	0.173	0.385***	0.654***					
9. Have the child fill in words at the end of a phrase	0.084	0.083	0.246*	0.681***	0.235*	0.368***	0.417***	0.310**				
10. Do an activity based on the story	0.012	0.344***	0.051	0.562***	0.241*	0.145	0.218*	0.243*	0.347***			
11. Practice rhyming	0.227*	0.153	0.233*	0.677***	0.342***	0.253**	0.369***	0.424***	0.325***	0.397***		
12. Parental self-efficacy	0.314***	0.004	0.465***	0.276**	0.103	0.371***	0.325***	0.159	0.208*	-0.085	0.158	
13. SES (composite)	0.165	-0.213*	0.351***	0.082	-0.017	0.288**	0.200*	0.078	0.125	-0.382***	0.001	0.443***

Note: *$p < 0.05$, **$p < 0.01$, ***$p < 0.001$. All correlations are bivariate Spearman's rho rank-order correlations, and all p values reflected are after FDR correction.
